# Acute Myeloid Leukemia: Updates on Diagnosis, Treatment and Management

**DOI:** 10.3390/cancers17142387

**Published:** 2025-07-18

**Authors:** Francesco Lanza, Michela Rondoni, Giovanni Marconi

**Affiliations:** Hematology Unit, Ravenna Hospital, University of Bologna, 48121 Ravenna, Italy; michela.rondoni@auslromagna.it (M.R.); giovanni.marconi@unibo.it (G.M.)

## 1. Introduction

This Special Issue of *Cancers*, entitled “Acute Myeloid Leukemia (AML): Updates on Diagnosis, Treatment and Management”, will be a forum for stimulating discussions and thought-provoking debates, featuring cutting-edge scientific manuscripts on the most relevant topics related to the diagnosis and therapeutic advances for the management of AML. This Special Issue will also provide invaluable opportunities to read articles from leading scientists who are shaping the future of AML research and treatment.

This editorial will attempt to highlight key issues and crucial developments in these areas of AML.

It is well known that AML is a clonal disorder resulting from acquired somatic mutations in hematopoietic progenitor cells that leads to the dysregulation of proliferation and differentiation of the hematopoietic system [1,2,3]. In recent years, many scientific publications have reported a huge number of genomic alterations, which can be used for AML classification [2,3,4,5,6,7,8]. Furthermore, the identification of genetic lesions is playing an increasing role in the prognosis and treatment of AML patients [4,5,6,7]. From a diagnostic point of view, the routine application of next-generation sequencing (NGS) as well as whole genome sequencing (WGS) into clinical practice has allowed better risk stratification of AML patients [2,3,4,5,6,7,8]. The most commonly altered genes include *FLT3*, *NPM1*, *DNMT3A*, *IDH1*, *IDH2*, *TET2*, *RUNX1*, *NRAS*, and *TP53* [4,5,6,7]. The incidence of these aberrations varies by patient age, previous exposure to chemotherapy and/or radiotherapy, and history of antecedent hematologic cancer. Accurate risk stratification and prognostication play a fundamental role in the management of AML patients, since it is well known that clinical outcomes vary widely for patients belonging to different cytogenetics and molecular-biology-based subcategories. These genetic risk subgroups are now being used for therapeutic guidance [4,5,6].

European LeukemiaNet (ELN) genetic risk classification is a reference document for the hematology community, and is widely used in either clinical practice or clinical trials [4,5].

However, there is a limitation to the use of this classification, since it is based exclusively on data from AML patients who have been receiving intensive chemotherapy, and cannot be applied in unfit/frail and/or older patients receiving less-intensive therapeutic approaches, especially for those individuals who belong to adverse risk categories [7]. Based on these considerations, we do believe that a novel genetic classification is needed for a correct prognostic stratification of patients receiving less-intensive treatment modalities, which may include hypomethylating agent (HMA, azacitidine (AZA) or decitabine)-based regimens alone or in combination with the B-cell leukemia/lymphoma 2 inhibitor venetoclax (VEN), or with the IDH1inhibitor ivosidenib (IVO) for the IDH1-mutated AML subgroup [9,10,11,12,13,14,15,16,17,18,19,20,21,22,23,24]. Preliminary efforts conducted at the Mayo Clinic [25] and by ELN authors [26] are based on a relatively low number of patients and have consequently already been demonstrated to require several refinements [27]. As far as the treatment scenario for AML is concerned, it must be pointed out that the treatment landscape for this disease is rapidly evolving, with novel mono- and combination therapies approved in the last decade [4,5,6,27,28].

Unfortunately, the prognosis for AML patients with high-risk genetic subsets remains unfavorable, and the development of more active treatment options for these patients is highly demanding.

High-risk AML is a heterogeneous group which includes therapy-related AML, AML originating from myelodysplastic syndrome (MDS) or myeloid neoplasms, and AML carrying a complex karyotype or multiple genetic lesions associated with worse prognosis [2,3,4,5,29,30,31].

The most relevant high-risk AML patient categories include (1) *Tp53*-mutated AML, (2) AML harbouring rearrangements involving the *KMT2A* locus, (3) secondary AML and (4) AML with a complex karyotype or carrying a chromosome and/or genetic lesions associated with poor outcomes (monosomal karyotype, −5 or del(5q); −7; −17/abn(17p), t(3q26.2;v)/*MECOM(EVI1)*-rearranged, inv(3)(q21.3q26.2) or t(3;3)(q21.3;q26.2)/*GATA2*, *MECOM(EVI1)*, t(8;16)(p11;p13)/*KAT6A*::*CREBBP*, t(9;22)(q34.1;q11.2)/*BCR*::*ABL1*, t(6;9)(p23;q34.1)/*DEK*::*NUP214)* [4,5].

## 2. TP53 AML

### 2.1. Biological Mechanisms Beyond This Special Entity

Mutations in TP53 disrupt the normal function of p53, the “guardian of the genome”, leading to loss of cell-cycle control, impaired DNA repair, and apoptotic escape in hematopoietic stem/progenitor cells. In AML, TP53 alterations often result in biallelic inactivation (e.g., TP53 mutation with 17p deletion or multiple TP53 hits), conferring a particularly aggressive phenotype [31,32,33,34]. Mechanistically, TP53 mutations can exert loss-of-function (LOF) effects by eliminating wild-type p53 activity, as well as dominant-negative effects, wherein a mutant p53 protein inhibits the remaining wild-type protein. Notably, certain missense mutations also have potential gain-of-function (GOF) properties—for example, aberrant p53 mutants can co-opt chromatin-modifying enzymes (such as EZH2) to promote leukemic self-renewal [34]. Preclinical models illustrate that hematopoietic stem cells (HSCs) bearing a TP53 missense mutation gain a competitive fitness advantage over HSCs with only monoallelic TP53 loss, yet are outcompeted by cells with complete TP53 loss. This hierarchy suggests that residual wild-type p53 activity imposes a dose-dependent tumor suppressor effect (biallelic loss > missense > monoallelic), affecting clonal fitness [35,36,37,38].

TP53-mutated AML frequently arises from antecedent clonal hematopoiesis or myelodysplasia. Although TP53-mutant clones have a relatively low intrinsic proliferative fitness in steady-state hematopoiesis—especially compared to common CHIP mutations such as DNMT3A—they possess marked resilience against cellular stresses. Cytotoxic exposures—notably chemotherapy and radiation—act as evolutionary bottlenecks that selectively favor the expansion of TP53-mutant clones present at low frequency prior to treatment. This phenomenon is evident in therapy-related AML, where the malignant clone can often be traced back to a preexisting TP53-mutated population that survived initial cancer therapy. Indeed, TP53 mutations are enriched in therapy-related myeloid neoplasms and bone marrow failure conditions, indicating that genotoxic stressors allow these mutant clones to outcompete normal HSCs. In summary, the loss of p53 confers a survival advantage under selective pressures (DNA damage, immune clearance), facilitating clonal evolution from pre-cancerous stages to frank leukemia [36,38].

A final striking aspect of TP53-mutated AML is an immunosuppressive tumor microenvironment. TP53-mutant leukemic stem cells upregulate immune checkpoint proteins like PD-L1, driving T-cell exhaustion and permitting immune escape [39]. In myelodysplastic syndromes (MDSs) and secondary AML, TP53 mutations confer an immune-evasive phenotype characterized by high PD-L1 expression on stem/progenitor cells, expansion of immunosuppressive regulatory T cells and myeloid-derived suppressor cells, and a paucity of cytotoxic T lymphocytes [40].

### 2.2. Clinical Outcomes and Prognostic Implications

Clinically, TP53-mutated AML offers one of the poorest prognoses in adult leukemia. Unlike many other AML subtypes, outcomes for TP53-mutant cases have not improved despite new therapies, with a median overall survival of approximately 6 months, even with intensive treatment, regardless of patient age or fitness status [37,41,42,43,44,45]. This dismal outcome reflects a high rate of primary chemo-refractoriness and early relapse. A recent analysis of frontline trials confirmed that median survival remained uniformly short across therapeutic approaches (chemotherapy, hypomethylating agents, venetoclax-based regimens), highlighting intrinsic chemoresistance in TP53-mutant AML [37].

Current research has focused on whether specific features of TP53 mutations modify this grim prognosis, specifically focusing on the burden of mutations and bi-allelic inactivation. Within a variety of results and thresholds, at the moment such speculation seems more relevant for MDS than for AML [32,37].

### 2.3. Therapies of TP53 Mutant AML

The therapeutic management of TP53-mutated AML remains challenging and is an area of active research. Standard cytotoxic chemotherapy (e.g., 7 + 3 regimen or CPX-351) yields complete remission rates of only approximately 30–40%, with short-lived remissions (median relapse-free survival of 3–6 months), and overall survival similar to less intensive approaches [37]. Venetoclax combined with hypomethylating agents (HMAs) has become standard for older or unfit patients; however, TP53-mutant AML demonstrates limited benefit. While venetoclax-based combinations induce initial responses, retrospective analyses indicate no significant survival advantage compared to HMA monotherapy [46,47,48].

Multiple novel agents have been explored to improve outcomes in TP53-mutated AML, but the parade of promising drugs that have not held up in later-phase studies has OK curbed progress, including with strategies to restore p53 function (Eprenetapopt combined with venetoclax) [49], bispecific antibodies [50], and agents to target pathways of immune evasion [51].

Allogeneic hematopoietic cell transplantation is, at the moment, the only treatment that offers chances for durable remission, even if the median survival of this population remains dismal (~15–20% at 2 years) [47,48,49,50,51,52].

Due to these biological considerations and poor clinical outcomes, the scientific community is reflecting on considering the TP53 mutant myeloid neoplasms as an entity [53,54].

## 3. *KMT2A*-rAML

AML carrying a rearrangement of *KMT2A* has been found to be associated with poor outcomes, with current treatment approaches not effective for treating patients with relapsed/refractory AML [55,56]. Recent data have documented that several types of AML have overexpression of *HOXA/B* cluster genes and *MEIS1*, which are key regulators of stem cell self-renewal and differentiation [57,58,59]. These can be dysregulated when aberrations of histone lysine *N*-methyltransferase 2A (KMT2A, formerly called “mixed-lineage leukemia fusion 1” (MLL1) and/or *NPM1* mutations occur [55,56]. Interestingly, the cytoplasmic localization of the mutant NPM1 is associated with a similar phenotype and genetic signature compared to KMT2A-altered AML.

The inhibition of the protein menin, (KMT2Ar;) and the mutated nucleophosmin gene (NPM1c), leading to the activation of transcription factors such as HOXA and MEIS1, is an innovative target therapy currently under investigation [55,56,57]. KMT2Ar is detected in 10% of AML cases, with a higher prevalence in younger patients who respond poorly to standard therapy [4,5]. NPM1 mutations are prevalent in AML patients and are mostly associated with a favorable prognosis. Comparable to KMT2Ar, NPM1c AML is characterized by the overexpression of HOXA/HOXB and menin, while a blockage of the MLL–menin complex can reinduce differentiation, leading to the inclusion of these patients in ongoing clinical trials using menin inhibitors [56,60]. Various menin inhibitors have been developed and investigated in pre-clinical and clinical trials; the use of revumenib (SNDX-5613) and ziftomenib has been associated with significant complete remission rates (30%) in relapsed/refractory (r/r) AML patients harboring KMT2Ar or NPM1c. Interestingly, menin inhibition was found to be associated with a substantial downregulation of key mediators of the MLL–menin complex, such as HOXA9, PBX3, CDK6 and MEIS1, thus reducing anti-apoptotic signaling molecules like BCL-2 and FLT3 and promoting the differentiation of AML cells.

Based on current investigations, we can speculate that in the near future, most of these patients will be treated successfully with menin inhibitors.

## 4. Secondary AML (sAML)

sAML is a heterogeneous group of AML, including therapy-related AML, AML originating from myelodysplastic syndrome or chronic myeloid neoplasms, and AML carrying genetic lesions associated with worse prognosis [61].

sAML presents as a complex of disorders, sometimes evolving from germline predisposing mutations, or the sequelae of cytotoxic therapies, and its development is driven by intricate genetic and epigenetic modifications [4,5,56]. Nowadays, genetic lesions are the hallmark of this entity. According to ICC and ELN classifications, the recognition of sAML is based exclusively on the presence of genetic mutations, such as aberrations in SRSF2, SF3B1, U2AF1, ZRSR2, ASXL1, EZH2, BCOR, or STAG2 genes [4,5]. Although the importance of prior therapy, antecedent myeloid neoplasms (i.e., MDS or MDS/MPN), and the development of underlying germline genetic disorders predisposed to AML is well recognized, current classifications now identify such associations as qualifiers for diagnosis rather than as specific disease categories [5]. Using this approach, the prior stand-alone categories of therapy-related myeloid neoplasms and AML with myelodysplasia-related changes are eliminated—genetic lesions are the hallmark of this entity.

The immunophenotypic profiles of these disorders are variable; however, accumulating data seem to support the role played by flow cytometry in the recognition of some of these varieties. This complexity requires a spectrum of therapeutic strategies, each meticulously tailored to address the distinct genetic profiles. Such strategies require a personalized approach, considering the heterogeneous clinical manifestations and biological characteristics of the leukemia clones [62,63,64].

According to ICC Classification, sAML encompasses both therapy-related myeloid neoplasms and antecedent MDS and/or myeloproliferative neoplasms, and are characterized by unfavorable outcomes. Allogeneic stem cell transplantation (HSCT) is a potentially curative approach for high-risk AML. HSCT should be used in combination with innovative treatment modalities such as CPX-351, venetoclax, tamibarotene, tamibarotene, and glasdegib (among many others), which have demonstrated promising results in enhancing prognosis and survival [65,66,67]. The evolving landscape of sAML treatment underscores the importance of continued research and innovation in the field, aiming not only to improve patient outcomes but also to enhance our knowledge of AML, thus allowing the application of more effective and personalized therapies. Based on this consideration, it can be stated that sAML represents a heterogeneous group of leukemias often associated with diverse immunophenotypic and genetic characteristics but inferior outcomes and survival.

Favorable-risk sAML is rare but can be managed similarly to de novo favorable-risk disease.

Furthermore, it must be pointed out that patients with *FLT3*—ITD-mutated AML continue to have suboptimal outcomes with standard therapies and experience high rates of relapse following HSCT. However, according to current AML classification, these entities are placed either in the intermediate or favorable group [67,68,69,70,71,72].

Another interesting area of investigation in this field is represented by artificial intelligence (AI). In recent years, cytogenetics and molecular biology studies have shown an impressive capacity to accurately define disease biology, with relevant prognostic implications. So far, AI–based algorithms have mainly focused on learning morphologic features that are associated with leukemia diagnosis. In oncology, these algorithms have been used to improve the current risk stratification capabilities of non-genitourinary cancers, colorectal cancer and cholangio carcinoma. Moreover, these algorithms have successfully been used to predict which patients may respond to standard-of-care therapies in several solid tumors. Furthermore, in the hematology field, preliminary investigations have demonstrated that the development of an AI-based predictive model may improve the prediction of post-transplant relapse in patients with AML receiving induction chemotherapy followed by HSCT, thus providing an effective tool for the early prediction and timely management of post-transplant relapse [73]. It is conceivable that this tool could soon aid hematologists in achieving improved and more precise prognosis and therapy for AML.
